# Synergistic effects of bioactive silica and fluoride in enamel protection and repair: an *in vitro* study

**DOI:** 10.1590/0103-644020256847

**Published:** 2026-01-12

**Authors:** Juliellen Luiz da Cunha, Anderson Gomes Forte, Elizabeth Barreto Galvão de Souza, Marcel Alves Avelino de Paiva, Adriana Moreira Ferreira, Rebeca Tibau Aguiar Dias, Fábio Correia Sampaio, Andressa Feitosa Bezerra de Oliveira

**Affiliations:** 1 Graduate Program in Dentistry, Paraíba Federal University(UFPB), João Pessoa, Paraíba, Brazil; 2 Graduate Program in Dental Materials, University of Campinas (UNICAMP), Piracicaba, São Paulo, Brazil; 3 School of Dentistry, Institutional Scientific Initiation Scholarship Program, Paraíba Federal University (UFPB), João Pessoa, Paraíba, Brazil; 4 Department of Chemical Engineering, Technology Center, Paraíba Federal University(UFPB), João Pessoa, Paraíba, Brazil; 5 Department of Clinical and Social Dentistry, Paraíba Federal University (UFPB), João Pessoa, Paraíba, Brazil; 6 Department of Morphology, Paraíba Federal University (UFPB), João Pessoa, Paraíba, Brazil

**Keywords:** Bioactive silica, Enamel remineralization, Fluoride, In vitro study, Tooth erosion

## Abstract

Erosive tooth wear (ETW) compromises enamel integrity. This study investigated the synergistic effects of bioactive silica and fluoride, as well as the independent performance of bioactive silica, in protecting sound enamel and repairing previously eroded enamel under erosive-abrasive challenges. An in vitro laboratory design was adopted using two models: (1) a protective model with sound enamel and (2) a remineralizing model with pre-eroded enamel. Ninety-six bovine enamel blocks were randomly allocated to four dentifrice groups (n=12/group) within two experimental models: RGS/NaF (bioactive silica + 1100 ppm NaF), RGS (bioactive silica without fluoride), NaF (1100 ppm NaF), and NC (fluoride/silica-free). Specimens were exposed to erosive cycling with 0.1% citric acid and simulated toothbrushing treatment for seven days (protective model) or five days (remineralizing model). Surface microhardness, quantitative light-induced fluorescence (QLF), 3D profilometry, and roughness were evaluated. Data were analyzed with ANOVA and Tukey’s post hoc test (p < 0.05). In the protective model, all groups showed microhardness loss, but RGS/NaF maintained the highest hardness values (p < 0.05). In the remineralizing model, all groups demonstrated partial recovery, with RGS/NaF significantly outperforming the others. Bioactive silica alone performed similarly to NaF in several parameters, showing reduced roughness and less structural loss compared with the negative control. The combination of bioactive silica and fluoride provided superior protective and remineralizing effects against erosive-abrasive enamel loss. Bioactive silica alone also exhibited relevant benefits, reinforcing its potential as a viable fluoride-free alternative.

## Resumo

O desgaste dentário erosivo (DDE) compromete a integridade do esmalte. Este estudo avaliou os efeitos sinérgicos da sílica bioativa e do fluoreto, bem como o desempenho da sílica isolada, na proteção do esmalte hígido e na recuperação do esmalte previamente erodido sob desafios erosivo-abrasivos. rata-se de um estudo in vitro com dois modelos experimentais: (1) protetor (esmalte hígido) e (2) remineralizante (esmalte previamente erodido). Noventa e seis blocos de esmalte bovino foram distribuídos em quatro grupos (n=12/grupo): RGS/NaF (sílica bioativa + 1100 ppm NaF), RGS (sílica bioativa sem fluoreto), NaF (1100 ppm NaF) e NC (sem flúor/sílica). As amostras foram submetidas à ciclagem erosiva com ácido cítrico a 0,1% e escovação simulada por sete dias (modelo protetor) ou cinco dias (remineralizante). Avaliaram-se microdureza, fluorescência quantitativa induzida por luz (QLF), perfilometria 3D e rugosidade. Os dados foram analisados por ANOVA e teste de Tukey (p < 0,05). No modelo protetor, todos os grupos apresentaram perda de microdureza, mas o RGS/NaF manteve os maiores valores (p < 0,05). No modelo remineralizante, todos mostraram recuperação parcial, com desempenho superior do RGS/NaF. A sílica bioativa isolada apresentou resultados semelhantes ao NaF em vários parâmetros, reduzindo rugosidade e perda estrutural em relação ao controle negativo. A combinação de sílica bioativa e fluoreto proporcionou efeitos protetores e remineralizantes superiores contra a perda erosivo-abrasiva do esmalte. A sílica bioativa isolada também mostrou benefícios relevantes, reforçando seu potencial como alternativa sem fluoreto.

**Figure:**
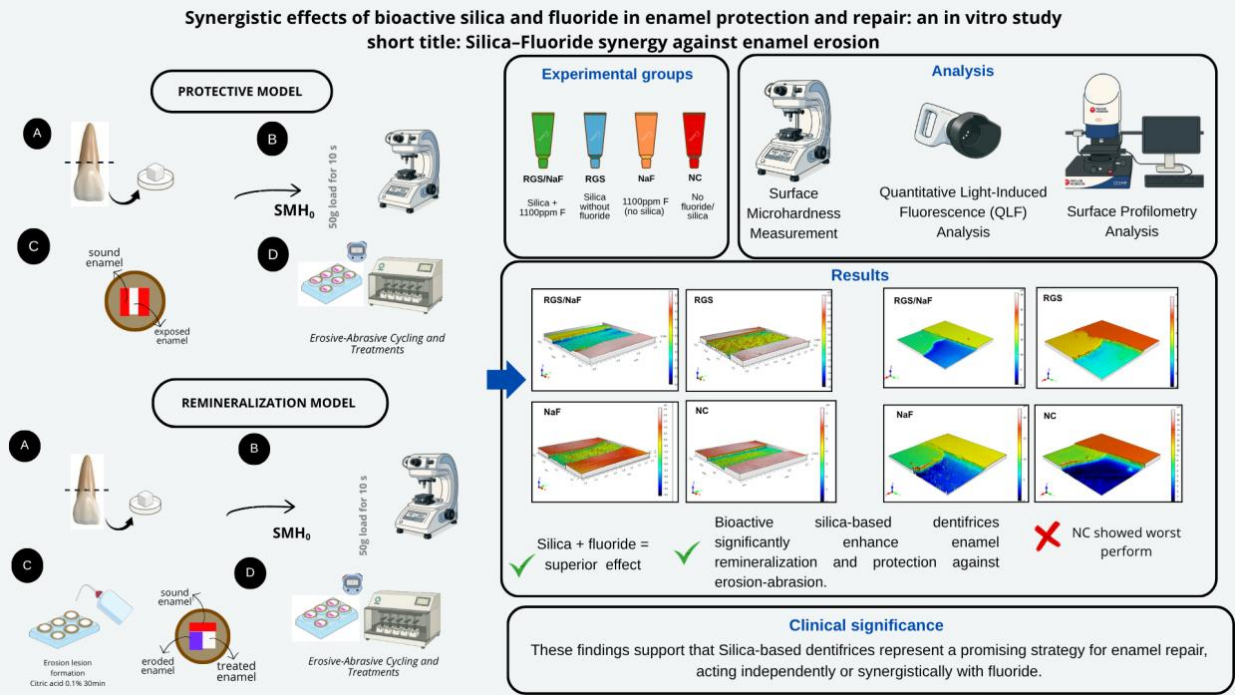

## Introduction

The exposure of dental enamel to acids of extrinsic or intrinsic origin promotes surface mineral softening, increasing its susceptibility to subsequent mechanical removal ^1^ ^,^ ^2^. This initial demineralization results in reduced microhardness and increased roughness, producing the characteristic “honeycomb” surface pattern ^3^ ^,^ ^4^. When brushing occurs on this softened substrate, the demineralized layer is rapidly removed, accelerating structural loss ^5^. This phenomenon defines erosive tooth wear (ETW), a condition caused by the combined action of non-bacterial acids and abrasion, leading to progressive changes in hardness, morphology, roughness, and mineral content ^6^ ^,^ ^7^.

The severity and progression of ETW depend on the enamel condition ^4^. Sound surfaces retain a protective aprismatic layer and perikymata, which are more mineralized and resistant to dissolution ^4^ ^,^ ^8^. Once this protection is lost, the underlying prismatic enamel, composed of more porous and soluble carbonated hydroxyapatite, is rapidly affected ^4^. This explains why previously eroded enamel recovers less effectively than intact enamel, even when exposed to conventional therapies ^9^.

Dentifrices are the primary non-invasive strategy for ETW management ^10^. Although fluoride formulations are widely used to inhibit demineralization, their efficacy against ETW remains limited, particularly on already eroded surfaces ^11^ ^,^ ^12^. Therefore, combining fluoride with bioactive agents has emerged as a promising approach to improve both protection and repair ^13^ ^,^ ^14^.

Silica, traditionally used as a mild abrasive and stain removal, has been structurally modified to acquire bioactive properties ^15^ ^,^ ^16^ ^,^ ^17^. Bioactive silica interacts with calcium and phosphate ions in saliva, promoting hydroxyapatite nucleation and forming a mineralized layer over demineralized enamel ^18^ ^,^ ^19^. This innovation expands the therapeutic role of dentifrices, supporting management of erosive-abrasive lesions, especially in early stages.

Different bioactive technologies have been incorporated into dentifrices ^15^ ^,^ ^16^. REFIX®, composed of silica and phosphate, favors the formation of a silica-enriched mineral layer resistant to erosive-abrasive challenges ^17^ ^,^ ^18^ ^,^ ^20^. NovaMin®, a calcium sodium phosphosilicate bioactive glass, releases calcium and phosphate that precipitate as hydroxycarbonate apatite and is mainly indicated for dentin hypersensitivity ^17^ ^,^ ^19^. Although both materials are bioactive, their mechanisms differ, and REFIX® shows greater potential for ETW management, which justifies further investigation.

Despite the clinical relevance of ETW, evidence regarding dentifrices containing bioactive silica, especially formulations without fluoride or directly compared to fluoride dentifrices, remains limited. Most available studies focus exclusively on sound enamel, whereas few explore previously eroded surfaces under erosive-abrasive challenges. This gap limits the understanding of both preventive and reparative effectiveness.

Previous studies investigating fluoride and calcium-phosphate systems, such as NovaMin® ^19^ ^,^ ^21^, have shown restricted performance when acid exposure is combined with brushing, particularly after erosion is already established. In contrast, little is known about the behavior of bioactive silica dentifrices under the same conditions, although initial findings suggest their potential to release ions and promote mineral deposition on softened enamel.

Considering these limitations, the present study advances current knowledge by directly comparing dentifrices containing bioactive silica (REFIX®), used alone or combined with fluoride, in standardized models simulating sound and pre-eroded enamel. To our knowledge, this is the first study to assess the silica-fluoride association through multiple complementary analyses, including microhardness, fluorescence, roughness, and surface loss.

Therefore, the aim of this study was to investigate the protective and remineralizing effects of bioactive silica dentifrices with or without fluoride under erosive-abrasive challenges. The null hypotheses stated that no significant differences would be observed among dentifrices for: ^1^ sound enamel subjected to seven days of erosive-abrasive cycling, and ^2^ previously eroded enamel subjected to five days of acid-abrasive challenge.

## Materials and methods

### Experimental Design

This in vitro laboratory study followed a randomized, comparative design with parametric statistical analysis. Two experimental models were conducted: ^1^ a protective model using sound enamel subjected to erosive-abrasive cycling, and ^2^ a remineralizing model using pre-eroded enamel. The same dentifrices and control products were tested in both models.

### Sample Size and Specimen Selection and Preparation

Bovine enamel blocks were selected as experimental substrates because their chemical composition, mineral content, and prismatic structure are comparable to human enamel, providing consistent mechanical and dissolution behavior under acid exposure and brushing. This substrate offers high standardization, reproducibility, and ethical availability, and is widely used in studies on erosive tooth wear and remineralization ^22^ ^,^ ^23^ ^,^ ^24^.

Sample size was calculated using the change in surface microhardness (ΔSMH) as the primary outcome. Based on a previous study ^25^, detecting a standardized difference of Cohen’s d = 1.29 with α = 0.05 and 80% power required nine specimens per group. To compensate for possible specimen loss and to preserve statistical power for multiple comparisons, the sample size was increased by ~30%, resulting in 12 specimens per group. Thus, each experimental model (protective and remineralizing) comprised four groups (n = 12), totaling 48 blocks per model and 96 enamel blocks overall.

Fifty freshly extracted bovine incisors were stored in 0.08% thymol solution for up to 30 days at room temperature. After cleaning, crowns were sectioned to obtain enamel blocks, and specimens were screened under a stereomicroscope (5×). Those exhibiting stains, cracks, or surface defects were excluded. Bovine teeth obtained as slaughterhouse by-products did not require ethical approval according to institutional and international standards.

Enamel blocks (≈ 4 × 4 × 2 mm) were obtained using a precision cutting machine (Labcut 1010, Extec) under continuous irrigation, embedded in acrylic resin, and planished with SiC papers of increasing grit under water. Final polishing was performed with felt disks and 1 µm diamond suspension before baseline assessments.

Surface microhardness (SH₀) was measured by three Vickers indentations (50 g, 10 s; HMV-G Series, Shimadzu, Japan) spaced 100 µm apart at the center of each specimen. Only samples within ±10% of the target mean (≈ 380 VHN) were included.

**Figure 1:**
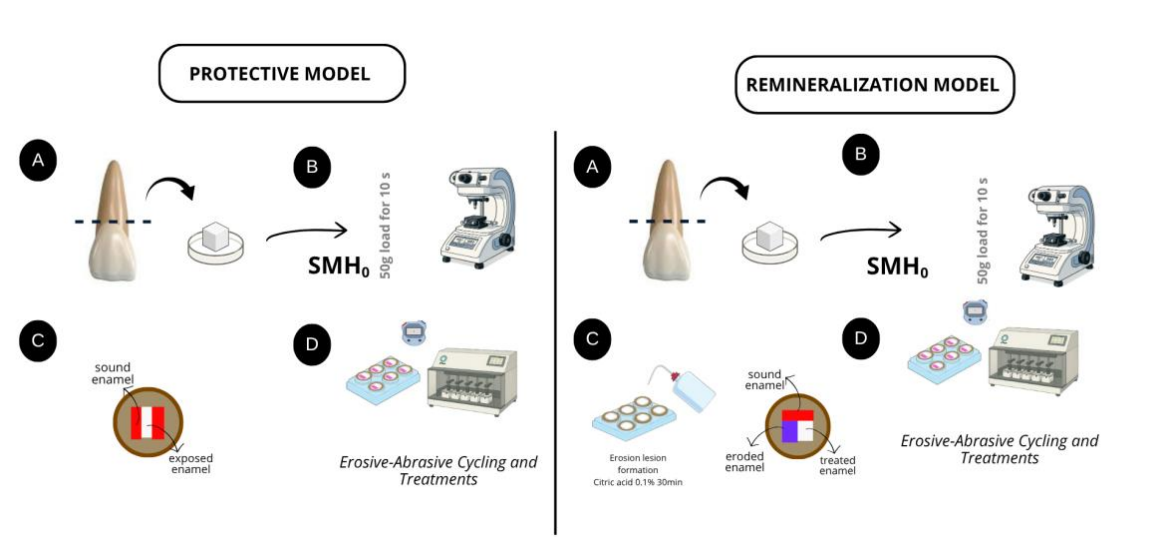
Schematic representation of the experimental design for the protective and remineralization models. Protective model: (a) enamel blocks obtained from bovine incisors, (b) baseline microhardness measurement, (c) surface division into control and test areas, (d) erosive-abrasive cycling and dentifrice treatment. Remineralization model: (a) enamel blocks obtained from bovine incisors, (b) baseline microhardness measurement, (c) induction of initial erosive lesion and surface division, (d) erosive-abrasive cycling and dentifrice treatment.

After baseline characterization, experimental regions were defined according to the model. In the protective model, each specimen was divided into three regions: two control areas at the ends, protected with acid-resistant nail varnish (Risqué, Goiania, Brazil), and a central exposed area subjected to erosive-abrasive cycling and dentifrice treatment. In the remineralization model, the surface was also divided into three regions: ^1^ sound control (protected before lesion formation), ^2^ eroded control (protected after lesion formation), and ^3^ test area (exposed to treatment). This intra-specimen design enabled direct comparisons between different conditions within the same sample (Figure 1).

### Erosive Lesion Formation

In the remineralization model, enamel lesions were artificially created prior to the experimental treatments. Each specimen was immersed in a 0.1% citric acid solution (pH ≈ 2.5) for 10 minutes under constant agitation (80 rpm) at room temperature. This protocol was based on previous studies demonstrating reproducible formation of erosive lesions with a softened surface layer ^22^ ^,^ ^23^. After demineralization, specimens were rinsed with deionized water, dried with absorbent paper, and the eroded control area was immediately protected with acid-resistant nail varnish to preserve the baseline reference (Figure 1).

Post-lesion microhardness (SH₁) was then measured using the same parameters applied for baseline SH₀. This step confirmed significant surface softening relative to baseline, validating the erosion protocol before the remineralization phase.

In the protective model, no erosive lesion was created. The central test area remained sound at baseline and was directly subjected to erosive-abrasive cycling.

### Dentifrice Selection and Slurry Preparation

Four dentifrices were tested (Table 1). To prevent allocation bias, all products were coded by an independent researcher, and both the operator and examiner were blinded to group identity until completion of the analyses.

Slurries were freshly prepared before each experimental cycle by diluting dentifrice in deionized water at a 1:3 (w/w) ratio (ISO 11609). Suspensions were homogenized immediately prior to use to ensure uniformity. During brushing, specimens were immersed in their respective slurries and treated under standardized abrasion conditions described in the Erosive-Abrasive Cycling and Treatment Protocols.

**Table 1:** Composition of the dentifrices used.

Dentifrice	Composition*	Manufacturer
Regenerating Sensitive Gel (RGS/NaF)	1100 ppm sodium fluoride, glycerin, silica, sorbitol, sodium lauryl sulfate, water, flavoring, PEF-12, cellulose gum, phosphoric acid, xylitol, tetrasodium pyrophosphate, sodium saccharin, triclosan, menthol, mica, sodium benzoate (REFIX® Technology)	Rabbit Corp, Londrina, PR, Brazil
Regenerating Sensitive Fluoride-Free Gel (RGS)	Fluoride-free formulation containing glycerin, silica, sorbitol, sodium lauryl sulfate, water, flavoring, PEF-12, cellulose gum, phosphoric acid, xylitol, tetrasodium pyrophosphate, sodium saccharin, triclosan, menthol, mica, and sodium benzoate (REFIX® Technology)	Rabbit Corp, Londrina, PR, Brazil
Fluoride Gel (NaF)	1100 ppm sodium fluoride, sorbitol, water, glycerin, silicon dioxide, sodium lauryl sulfate, sodium carmellose, flavoring, xylitol, sodium saccharin dihydrate, sodium benzoate	Rabbit Corp, Londrina, PR, Brazil
Negative Control Gel (NC)	Fluoride- and silica-free formulation containing glycerin, sorbitol, water, silica, cellulose gum, flavoring, xylitol, sodium saccharin, sodium benzoate	Rabbit Corp, Londrina, PR, Brazil

### Erosive-Abrasive Cycling and Treatments Protocols

### Protective Model (Sound Enamel)

This protocol, adapted from Simões et al. ^25^ and Buzalaf et al. ^13^, was conducted over seven consecutive days. Specimens were stored overnight in artificial saliva (pH 6.8) before cycling. From day 1 to 7, they underwent three daily erosive challenges, consisting of immersion in 0.1% citric acid (pH 2.5, 90 s, 37 °C), followed by rinsing with deionized water (5 s) and immersion in artificial saliva (30 mL/specimen). Artificial saliva was prepared according to Magalhães et al. ^26^ and contained 0.2 mM glucose, 9.9 mM NaCl, 1.5 mM CaCl₂·2H₂O, three mM NH₄Cl, 17 mM KCl, two mM NaSCN, 2.4 mM K₂HPO₄, 3.3 mM urea, 2.4 mM NaH₂PO₄, and 11 μM ascorbic acid. Proteins such as mucin were intentionally excluded, since they may interfere with pellicle formation and mineral dynamics. This solution was used in both experimental models.

After the first acid challenge each day, specimens remained in artificial saliva for 30 min before brushing, simulating the natural buffering and remineralizing effect of saliva. Brushing was performed in a mechanical brushing machine (MEV 3T-8XY, Odeme, Brazil) with soft-bristle toothbrushes (Colgate® Classic Clean), one per specimen, using freshly prepared slurry (dentifrice: deionized water, 1:3, 30 mL). Each brushing cycle consisted of 10 s of brushing followed by 110 s of slurry contact, under standardized conditions: 37 °C, frequency of 11 cycles/10 s, 25 mm vertical motion, and 150 g axial load. After brushing, specimens were rinsed with deionized water and re-immersed in artificial saliva. At the end of the third daily erosive challenge, the brushing protocol was repeated. Specimens were stored overnight in artificial saliva at 37 °C.

### Remineralization Model - Eroded Enamel

This protocol, adapted from Simões et al. ^25^, was conducted over five consecutive days at 37 °C. Specimens were stored overnight in artificial saliva (pH 6.8) prior to cycling. Each day, specimens were immersed in artificial saliva before and after the erosive challenges. Three daily erosive episodes were performed, consisting of immersion in 0.1% citric acid (pH 2.5, 90 s) under gentle agitation, followed by rinsing with deionized water (10 s) and immersion in artificial saliva (30 mL/specimen, 25 °C) for two hours. After the first and last acid exposures of each day, brushing was performed under the same conditions described for the protective model. Overnight, specimens remained in artificial saliva at 37 °C.

All solutions were prepared in advance and renewed daily.

### Surface Microhardness Analysis

Surface microhardness was evaluated at each experimental stage using the parameters previously described.

In the protective model, baseline hardness (SH₀) was measured before the cycling, and final surface hardness (SH₁) was determined after completion of the erosive-abrasive protocol and dentifrice treatments. The percentage of surface hardness change (%SMHC) was calculated as: *%SMHC = [(SH₀ - SH₁) / SH₀] × 100.*

In the remineralization model, baseline (SH₀) and post-lesion (SH₁) values were first recorded, and final hardness (SH₂) was determined after the erosive-abrasive cycling with dentifrice treatments. The percentage of surface hardness recovery (%SMHR) was calculated as: *%SMHR = [(SH₂ - SH₁) / (SH₀ - SH₁)] × 100.*

### Quantitative Light-Induced Fluorescence (QLF) Analysis

Mineral loss was assessed using the Qraycam Pro device (Inspektor Research Systems BV, Amsterdam, Netherlands). Prior to imaging, the acid-resistant varnish was carefully removed using cotton swabs dampened with acetone. Specimens were rinsed with deionized water, air-dried, and imaged in a darkened environment at a fixed distance of 8 cm between the device and the specimen. Exposure and contrast settings were standardized at zero for all measurements. Image analysis was performed using Q-ray software (version 1.38), which quantified mineral loss through fluorescence reduction (ΔF). The parameter ΔFmax corresponded to the highest fluorescence loss detected, indicating the maximum depth of demineralization.

In the protective model, ΔF values were calculated by comparing the sound control area to the treated area, reflecting the fluorescence loss and lesion depth induced by erosive-abrasive cycling.

In the remineralization model, two measurements were obtained: ^1^ ΔF₀, the fluorescence loss difference between the sound and eroded areas at baseline (pre-treatment), and ^2^ ΔF₁, the difference between the sound and treated areas after cycling (post-treatment). The variation in fluorescence was expressed as: *ΔFdif = ΔF₁ - ΔF₀*. Positive ΔFdif values indicated mineral gain after treatment, while negative values indicated additional mineral loss ^23^. The same procedure was applied to ΔFmax, producing ΔFmax₀, ΔFmax₁, and the variation ΔFmaxdif.

### Profilometry Analysis

Surface morphology and enamel loss were evaluated using a three-dimensional non-contact optical profilometer (Talysurf CCI MP, Taylor Hobson, Leicester, UK). The instrument operates by coherence scanning interferometry, capturing enamel topography along the x, y, and z axes with nanometric vertical resolution. Scans were obtained at 20× magnification over a field of view of 0.86 × 0.86 mm² in XY scanning mode (1024 × 1024 pixels) and processed using a Gaussian filter with a 0.25 mm cut-off, in accordance with ISO 16610-61. The profilometer generates three-dimensional height maps of the scanned area and quantifies the vertical distance between the reference (sound) and eroded or treated regions. The mean height difference is expressed as Surface Loss (SL, µm), representing enamel loss after erosive-abrasive challenges.

In the protective model, surface roughness (Ra) was measured in the sound region (Ra₀) and in the treated area (Ra₁), and roughness variation was calculated as ΔRa = Ra₁ − Ra₀. In the remineralization model, Ra was measured in three regions: sound enamel at baseline (Ra₀), eroded enamel after acid exposure and before treatment (Ra₁), and treated enamel after cycling (Ra₂), with roughness variation calculated as ΔRa = Ra₂ − Ra₁. Structural loss was also quantified through the SL parameter. In the remineralization model, SL₀ represented the difference between sound and eroded areas before treatment, while SL₁ represented the difference between sound and treated areas after cycling. The effect of treatment was calculated as ΔSL = SL₁ − SL₀, where positive values indicate additional surface loss and negative values indicate partial surface recovery.

All profilometric measurements were obtained at three longitudinal positions per specimen (25%, 50%, and 75% of the surface width), and the arithmetic mean was used for statistical analysis. Three-dimensional reconstructions generated by the profilometer were employed qualitatively to illustrate morphological differences among groups and corroborate the quantitative SL values ^24^.

### Data Analysis

Data were analyzed using SPSS software (version 21.0, SPSS Inc., Chicago, IL, USA). Normality was verified with the Shapiro-Wilk test and homogeneity of variances with Levene’s test. As both assumptions were satisfied, no data transformation was required.

Intergroup comparisons were performed using one-way ANOVA followed by Tukey’s post hoc test for the variables: surface microhardness (SH), percentage of surface hardness change (%SMHC), percentage of surface hardness recovery (%SMHR), fluorescence loss (ΔF, ΔFmax), surface roughness (Ra), and surface loss (SL).

Intragroup comparisons were carried out using paired t-tests for SH₀ vs. SH₁ and Ra₀ vs. Ra₁ in the protective model. In the remineralization model, repeated measures ANOVA was applied for SH₀-SH₂ and Ra₀-Ra₂, with prior verification of sphericity.

The level of statistical significance was set at α = 0.05 for all tests.

## Results

### Surface Microhardness Analysis

In the protective model, after erosive-abrasive cycling and dentifrice treatments (SH₁), the RGS/NaF group maintained the highest surface hardness values, significantly different from all other groups (p < 0.05). The NaF and RGS groups showed intermediate hardness values with no significant difference between them (p > 0.05), while the NC group presented the lowest hardness (Table 2). The percentage of surface microhardness change (%SMHC) confirmed these results, with RGS/NaF showing the smallest loss, NaF and RGS intermediate values, and NC exhibiting the most significant decrease.

In the remineralization model, after lesion formation (SH₁) and subsequent cycling with treatments, the RGS/NaF group showed the highest final hardness (SH₂), significantly higher than all other groups (p < 0.05). Both RGS and NaF promoted partial recovery with similar performance, whereas NC demonstrated minimal improvement (Table 2). The percentage of surface microhardness recovery (%SMHR) reflected the same trend: RGS/NaF exhibited the most remarkable recovery, RGS and NaF exhibited intermediate effects, and NC exhibited the lowest recovery values.

**Table 2:** Means and standard deviations of surface microhardness (VHN) in the protective model [sound (SH₀) and treated (SH₁) areas] and in the remineralization model [sound (SH₀), eroded (SH₁), and treated (SH₂) areas].

Groups	Protective model			Remineralization model			
	SH_0_	SH_1_	%SMHC	SH_0_	SH_1_	SH_2_	%SMHR
RGS/NaF	387.9±2.0 ^a, A^	288.5±6.2 ^a, B^	26.9± 1.35^a^	390.0±6.6 ^a, A^	193.4±7.3 ^a, B^	334.2±13.2 ^a, C^	71.7± 6^a^
RGS	289.6 ±1.0 ^a, A^	250.5±5.7 ^b, B^	35.6 ± 1.51^b^	387.6±5.2 ^a, A^	190.7±67 ^a, B^	291.5±17.9 ^b, C^	51.2±9.3^b^
NaF	389.5±1.3 ^a,A^	221.5±7.8 ^b,B^	43.1 ± 5.65^b^	391.2±8.9 ^a, A^	193.3±7.4 ^a, B^	306.8±10.3 ^c, C^	58.1± 8.9^b^
NC	388.5±2.6 ^a, A^	160.2±4.5 ^c, B^	58.7± 2.89^c^	389.6±7.8 ^a, A^	192.9±6.5 ^a, B^	210.9±14 ^d, C^	8.9± 5.7^c^

*Lowercase letters compare groups within the same column. Uppercase letters compare conditions within the same row. Different letters indicate significant differences (p < 0.05). No comparisons were made between the two models.

### Quantitative Light-Induced Fluorescence (QLF) Analysis

**Figure 2:**
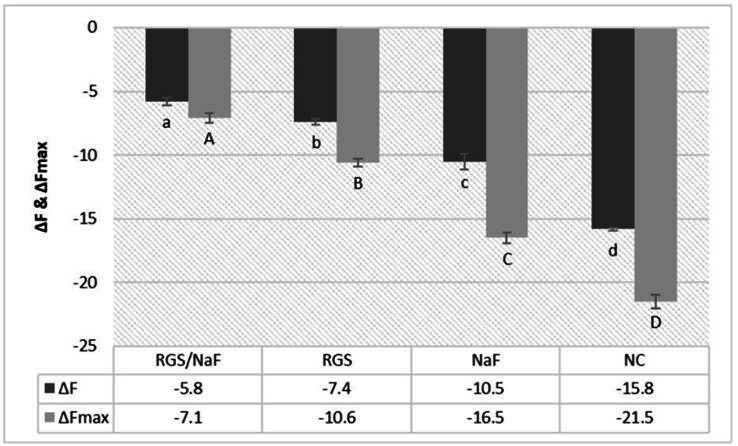
Enamel fluorescence values (ΔF and ΔFmax) in the protective model (mean ± SD). Lowercase letters indicate significant differences between groups (columns) for ΔF. Uppercase letters indicate significant differences between groups (columns) for ΔFmax (p < 0.05).

In the protective model, ΔF values showed that the RGS/NaF group experienced the lowest fluorescence loss, significantly different from all other groups (p < 0.05). The NaF and RGS groups presented intermediate fluorescence loss with no significant difference between them, while NC exhibited the highest ΔF (Table 3). The ΔFmax results followed the same pattern (Figure 2), indicating that RGS/NaF minimized both mean and maximum mineral loss when compared with the other groups.

In the remineralization model, baseline fluorescence loss between sound and eroded areas (ΔF₀) was confirmed, validating lesion formation. After cycling, the RGS/NaF and RGS groups showed significant mineral gain, reflected by positive ΔFdif values, with no significant difference between them (p > 0.05). The NaF promoted partial recovery, while NC presented negative ΔFdif values, indicating continued mineral loss (Table 3). ΔFmaxdif results showed similar trends: RGS/NaF and RGS produced greater reduction in maximum lesion depth, NaF demonstrated an intermediate effect, and NC exhibited worsening lesions.

**Table 3:** Means and standard deviations of enamel fluorescence values in the remineralization model: difference between sound and eroded areas (ΔF₀), sound and treated areas (ΔF₁), and their variation (ΔFdif); maximum fluorescence loss (ΔFmax₀, ΔFmax₁) and its variation (ΔFmaxdif).

Groups	ΔF_0_	ΔF_1_	**ΔF*dif***	ΔFmax_0_	ΔFmax_1_	**ΔF*difmax***
RGS/NaF	-12.7 ± 1.0^a,A^	-6.7 ± 0.6^a,B^	5.9 ± 1,1^a^	-22.6 ± 1.5^aA^	-10.2 ± 3.0^aB^	12.6 ± 2.4^a^
RGS	-13.0 ± 0.6^a,A^	-6.6 ± 0.5^a,B^	6.3 ± 0,8^a^	-23.0 ± 1.4^aA^	-9.5 ± 2.1^aB^	13.5 ± 2.1^a^
NaF	-12.8 ± 1.2^a,A^	-10.6 ± 0.7^b,B^	1.8 ± 0,4^b^	-23.7 ± 1.6^aA^	-17.7 ± 1.7^bB^	4.4 ± 0.6^b^
NC	-12.5 ± 0.8^a,A^	-14.7 ± 1.8^c,B^	-1.9 ± 0,9^c^	-22.2 ± 2.9^aA^	-30.2 ± 2.5^cB^	-6.5 ± 0.7^c^

*Lowercase letters compare groups within the same column. Uppercase letters compare conditions within the same row. Different letters indicate significant differences (p < 0.05).

### Surface Roughness and Enamel Surface Loss

**Figure 3:**
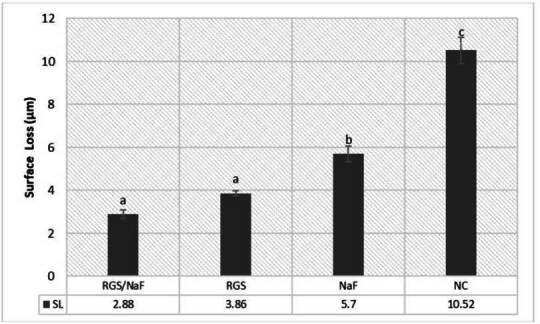
Enamel surface loss (SL) in the protective model (mean ± SD). Lowercase letters indicate significant differences between groups (columns) (p < 0.05).

In the protective model (Table 4), the RGS/NaF group showed the lowest increase in surface roughness (ΔRa), significantly different from the other groups (p < 0.05). The NaF and RGS groups presented intermediate ΔRa values with no significant difference between them, while NC exhibited the most tremendous increase. Similar results were observed for surface loss (SL), with RGS/NaF showing the most minor enamel loss, NaF and RGS intermediate performance, and NC the highest structural loss (Figure 3).

**Figure 4:**
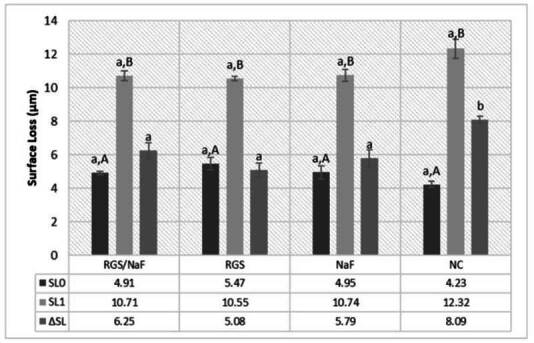
Surface loss between sound and eroded areas (SL₀), sound and treated areas (SL₁), and their variation (ΔSL) in the remineralization model (mean ± SD).Lowercase letters indicate significant differences between groups (columns). Uppercase letters indicate significant differences within groups (rows) (p < 0.05).

In the remineralization model (Table 4), RGS/NaF and RGS both promoted significant reductions in surface roughness (ΔRa) compared with the eroded baseline, with no significant difference between them (p > 0.05). NaF showed partial improvement, while NC presented the highest roughness values after cycling (Table 4). Regarding surface loss, RGS/NaF and RGS again achieved the lowest values, NaF intermediate, and NC the most significant enamel loss (Figure 4).

**Table 4:** Means and standard deviations of surface roughness parameters in the protective and remineralization models.

Groups	Protective model			Remineralization model			
	Ra₀	Ra₁	ΔRa	Ra₀	Ra₁	Ra₂	ΔRa
RGS/NaF	0.0361 ± 0.00 ^a,A^	0.1911± 0.02 ^a,B^	0.1550± 0.02 ^a^	0.0187± 0.01^a,A^	0.2311 ± 0.02^a,B^	0.1622 ± 0.01^a,C^	-0.0688 ± 0.02^a^
RGS	0.0296 ± 0.00^a, A^	0.2567± 0.09 ^a, B^	0.2271± 0.09 ^a^	0.0288± 0.00^a, A^	0.2544 ± 0.01^a, B^	0.1500 ± 0.05^a,C^	-0.1044 ± 0.01^a^
NaF	0.0390 ± 0.01^a, A^	0.7820± 0.03^b, B^	0.7430 ± 0.02 ^b^	0.0222± 0.01^a, A^	0.2666± 0.02^a, B^	0.2088 ± 0.02^a,B^	-0.0577 ± 0.02^a^
NC	0.0346 ± 0.01 ^a, A^	0.9520 ± 0.02 ^b, B^	0.9174 ± 0.02 ^b^	0.0188 ± 0.01 ^a, A^	0.2255 ± 0.02 ^a, B^	0.2822± 0.02^a,B^	0.0566± 0.01^b^

* Lowercase letters indicate significant differences between groups within the same column. Uppercase letters indicate significant differences within the same row (p < 0.05).

Overall, profilometry results demonstrated that RGS/NaF offered the best protective and reparative effects, while RGS and NaF showed moderate but significant benefits compared with NC.

### Qualitative Surface Topography

**Figure 5:**
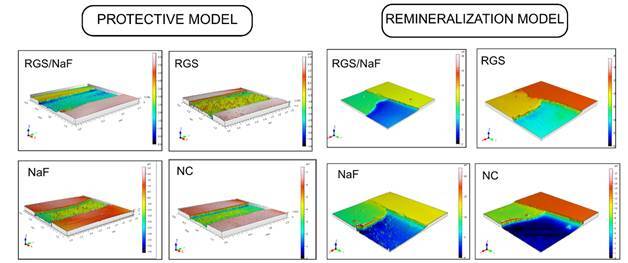
Representative 3D profilometric images of treated enamel surfaces, illustrating the effects of the different dentifrices on erosive-abrasive wear.

Representative 3D surface maps are shown in Figure 5.

In the protective model, NC specimens exhibited extensive structural degradation, with irregular topographies characterized by deep depressions, discontinuities, and pronounced roughness. NaF and RGS showed less severe alterations, with localized erosion and shallow surface irregularities. In contrast, RGS/NaF preserved a more uniform topography, displaying smoother contours and minimal surface disruption, indicating adequate protection against erosive-abrasive challenges.

In the remineralization model, NC revealed the most extensive defects, with marked depressions and heterogeneous surfaces, consistent with progressive mineral loss. NaF promoted partial improvement, producing surfaces with reduced but still evident irregularities. RGS and RGS/NaF exhibited flatter and more continuous topographies, with fewer defects and signs of surface filling and leveling, compatible with mineral deposition and repair of the previously eroded enamel.

Overall, the topographic images corroborate the quantitative profilometric findings. RGS/NaF most effectively limited surface degradation and promoted morphological recovery, whereas RGS and NaF produced intermediate effects compared with the untreated control.

## Discussion

The null hypotheses were rejected, since significant differences among dentifrices were observed in both experimental models. Overall, RGS/NaF consistently provided the most favorable outcomes, followed by RGS and NaF, while NC showed the least protective and remineralizing effects.

In the protective model, the superior performance of RGS/NaF was demonstrated by higher SH values and lower %SMHC, ΔF, ΔFmax, ΔRa, and SL compared with other groups. Rather than merely preventing surface loss, these results suggest a more stable mineral balance under repeated erosive-abrasive stress. This supports previous findings that fluoride alone provides limited protection under severe acid challenges, while bioactive agents enhance efficacy through complementary mechanisms ^13^ ^,^ ^21^. The fact that RGS also reduced mineral loss compared with NC, although not significantly different from NaF, reinforces the advantage of combining bioactive silica with fluoride to achieve consistent protective effects ^19^ ^,^ ^21^ ^,^ ^27^.

In the remineralization model, RGS/NaF again demonstrated the most favorable outcomes, with the highest recovery of hardness (%SMHR), mineral gain (ΔFdif, ΔFmaxdif), and improved surface parameters (ΔRa, ΔSL). These findings indicate that silica-fluoride synergism extends beyond prevention, contributing to effective repair of previously eroded enamel ^21^. RGS also promoted partial recovery, comparable to NaF in several parameters, confirming that bioactive silica can support remineralization, but less effectively than when combined with fluoride. The enhanced performance of RGS/NaF likely relates to greater formation of calcium fluoride and fluorapatite, which strengthens enamel against acidic and abrasive challenges ^10^ ^,^ ^28^.

Surface topography analyses supported these results. RGS/NaF exhibited less topographical degradation and signs of structural reorganization, indicating not only interruption of erosive progression but also potential restorative effects, which are clinically relevant. Oliveira et al. ^21^ demonstrated that bioactive silica-fluoride dentifrices can partially restore enamel by forming a mineralized surface layer even without brushing. The present findings expand on that evidence, showing that even under brushing conditions, which impose additional mechanical stress, bioactive formulations-particularly RGS/NaF-maintain significant efficacy.

Differences between models were expected due to substrate characteristics. Sound enamel, covered by an intact aprismatic layer, is more resistant to erosion and abrasion, delaying mineral loss ^4^ ^,^ ^8^. Once demineralization occurs, mineral density decreases, porosity increases, and the prismatic structure is exposed, making remineralization less effective and recovery values lower than those observed in the protective model ^9^ ^,^ ^29^. These findings reinforce the importance of early preventive strategies to minimize cumulative tissue loss.

Brushing intensified wear in the protective model but appeared to facilitate mineral retention in the remineralization model. Even under combined acid-abrasive stress, bioactive silica promoted remineralization, although with attenuated effects ^29^. Dentifrice composition was more decisive than brushing timing, as immediate or delayed brushing did not significantly affect outcomes ^30^ ^,^ ^31^. Even in the unfavorable condition of brushing immediately after acid exposure, RGS/NaF achieved significant improvements in hardness and fluorescence, reinforcing its effectiveness in complex erosion-abrasion scenarios.

The synergistic activity of bioactive silica and fluoride explains these outcomes. Fluoride favors the precipitation of fluorapatite or fluoridated hydroxyapatite, increasing resistance to acid ^12^. Bioactive silica contains silanol groups (Si-OH) that act as nucleation sites, facilitating calcium and phosphate deposition and accelerating the formation of fluoride-rich apatite ^13^ ^,^ ^18^ ^,^ ^19^. This mechanism leads to the formation of a dense, acid-resistant mineral layer, composed of enamel-like apatite and exhibiting morphological similarity to natural enamel, supporting the notion of proper structural repair rather than superficial rehardening ^13^ ^,^ ^14^ ^,^ ^18^ ^,^ ^19^ ^,^ ^20^. In addition, bioactive silica may function as a controlled ion-release system, modulating pH and supporting crystal growth ^17^ ^,^ ^18^ ^,^ ^27^. Silva et al. ^32^ observed that fluoride can replace hydroxyl at A sites and silica can replace phosphate at B sites, promoting silicon-enriched apatite. Parker et al. ^33^ reported that such substitutions generate a less soluble and more bioactive mineral phase, consistent with the durability observed in the present study. Taken together, these mechanisms indicate that bioactive silica not only provides ions for remineralization but also reorganizes enamel crystal structure, an effect that appears to be amplified when combined with fluoride ^34^ ^,^ ^35^.

Although RGS/NaF showed the best overall performance, RGS alone still benefited enamel, particularly by reducing roughness and surface loss. This suggests that bioactive silica dentifrices may serve as adjunctive strategies when fluoride use is limited or contraindicated.

Different silica-based technologies have distinct clinical indications. REFIX® combines silica and phosphate to form a durable silica-enriched mineral layer resistant to acid and abrasion ^17^ ^,^ ^18^ ^,^ ^20^. In contrast, NovaMin® primarily releases calcium and phosphate ions for hydroxycarbonate apatite deposition, being more associated with dentin hypersensitivity relief ^16^. Although NovaMin® can induce surface repair, its effects on enamel are less stable under erosive-abrasive challenges ^19^ ^,^ ^21^ ^,^ ^36^. The present findings reinforce the preventive potential of REFIX® formulations in the management of erosive tooth wear ^20^.

From a clinical perspective, these findings highlight that bioactive silica-fluoride dentifrices may help prevent and repair early stages of erosive tooth wear, improving surface hardness, mineral retention, smoothness, and resistance to acid and abrasion. Given the growing prevalence of ETW linked to diet and lifestyle ^6^ ^,^ ^7^ ^,^ ^11^, such formulations may contribute to maintaining enamel integrity, aesthetics, and sensitivity control. The erosive protocol used in this study (citric acid, pH 2.5, followed by brushing) simulates daily exposure to acidic beverages and toothbrushing soon after meals, representing early-to-moderate ETW stages, in which softened enamel undergoes cumulative mechanical loss. Therefore, the ability of bioactive silica and fluoride formulations, particularly RGS/NaF, to maintain hardness, fluorescence, and surface integrity under these conditions reinforces their clinical relevance in preventing and managing enamel erosion.

The mechanical and optical properties evaluated-microhardness, fluorescence, roughness, and surface loss-collectively demonstrate the clinical significance of the results. RGS/NaF produced significantly higher microhardness values, indicating a more compact and mechanically resistant mineral surface. Harder enamel better resists mechanical deformation and acid dissolution, reducing susceptibility to future erosive-abrasive episodes ^4^ ^,^ ^5^. The increase in fluorescence (ΔF) detected by QLF confirms subsurface remineralization and mineral retention promoted by the silica-fluoride interaction. Additionally, RGS/NaF produced lower surface roughness values (<0.2 µm), below the threshold for bacterial biofilm accumulation ^4^ ^,^ ^37^, reducing the potential for plaque retention and acid diffusion. The reduction in surface loss (SL) observed in the bioactive groups demonstrates preservation of enamel thickness, minimizing the risk of microstructural fatigue and the development of non-carious cervical lesions ^9^ ^,^ ^38^. Taken together, these findings indicate that the bioactive silica-fluoride interaction enhances the mechanical and chemical resilience of enamel.

A strength of this study is the controlled in vitro design, which ensures variable standardization, reproducibility, and helps clarify the mechanisms of action of bioactive agents ^13^. However, in vitro protocols cannot fully replicate the oral environment, pellicle formation, salivary proteins, pH fluctuations, or masticatory forces ^3^ ^,^ ^10^. As Buzalaf et al. ^13^ suggest, synergistic effects may be even more pronounced in vivo. Thus, further in situ and clinical trials are necessary to validate long-term benefits.

Finally, the dual-model approach simulated both sound and eroded enamel, representing preventive and therapeutic scenarios ^4^ ^,^ ^8^. This strengthens the clinical relevance of the findings and supports the role of bioactive dentifrices in preventive dentistry.

## Conclusion

The combination of bioactive silica and fluoride was the most effective approach in both models, reducing mineral loss in sound enamel and promoting superior recovery in previously eroded enamel. Bioactive silica alone also provided relevant benefits, preserving surface morphology and supporting partial repair, indicating potential as a fluoride-free alternative. Overall, these findings confirm the synergistic interaction between bioactive silica and fluoride and support the use of bioactive dentifrices as preventive agents for managing erosive tooth wear.
